# Functional consequences of age-related morphologic changes in pyramidal neurons of the rhesus monkey prefrontal cortex

**DOI:** 10.1186/1471-2202-14-S1-P412

**Published:** 2013-07-08

**Authors:** Patrick Coskren, Doron Kabaso, Susan L Wearne, Aniruddha Yadav, Patrick R Hof, Jennifer I Luebke, Christina M Weaver

**Affiliations:** 1 Fishberg Department of Neuroscience and Friedman Brain Institute, Icahn School of Medicine at Mount Sinai, New York, NY 10029, USA; 2 Department of Anatomy and Neurobiology, Boston University School of Medicine, Boston, MA 02118, USA; 3 Department of Mathematics, Franklin and Marshall College, Lancaster, PA 17604, USA

## 

In normal aging, neocortical pyramidal neuron dendrites and dendritic spines undergo significant changes [1,2], often with concomitant physiological changes. In layer 3 of the prefrontal cortex (PFC) of the rhesus monkey, aged pyramidal neurons have a significantly higher input resistance and higher action potential (AP) firing rates *in vitro *compared to young neurons [3]. Our multidimensional approach combines whole-cell patch clamp recording, confocal microscopy, 3D digital reconstruction, and computational modeling to explore structure/function relationships. We now have a unique database of electrophysiological recordings, morphologic reconstructions, and compartment models from six young and six aged layer 3 pyramidal neurons from the rhesus monkey PFC.

As in prior studies [4], the length of individual dendritic branches were significantly shorter in aged than in young neurons, with fewer dendritic spines. These morphological changes significantly reduced the somatofugal and somatopetal dendritic voltage attenuation in aged versus young model neurons. However, they were insufficient to account for the increase in input resistance observed *in vitro*, even after including synaptic background activity constrained by cell-specific total spine number. This suggests that specific membrane resistance (*R_m_*) is higher on average in aged neurons. Using our recently developed model [5], we conducted a systematic sampling of the parameter space of Hodgkin-Huxley maximal conductances for each of the twelve model neurons, to fit firing rates of each model to the mean young and aged rates recorded empirically. In both age groups, some model neurons had several good fits to empirical data, while others had no good fits; there was no difference in the number of best-fit parameter sets from young and aged models. When the same conductance parameters were applied to all models, the mean firing rates of young and aged model neurons did not differ. This result also held when different values of *R_m _*were applied to each age group, and when a wider array of voltage- and calcium-gated ion channels were included.

Overall these simulations predict that age-related morphologic differences do affect dendritic signal integration, but do not account for changes in neuronal excitability observed in *in vitro *recordings. Our modeling suggests that morphology, passive cable properties, and active channel conductances could trade off against one another, constraining neuronal excitability within a certain range for each age group. Even so, we predict that the membrane resistance and active channel conductances of PFC pyramidal cells are changed with aging. Such predictions begin to reveal how networks comprising these neurons may function differently in young and aged animals.
